# Multiscale Thermal Investigations of Graphite Doped Polystyrene Thermal Insulation

**DOI:** 10.3390/polym14081606

**Published:** 2022-04-14

**Authors:** Ákos Lakatos, Attila Csík

**Affiliations:** 1 Department of Building Services and Building Engineering, Faculty of Engineering, University of Debrecen, Ótemető Str 2-4, 4028 Debrecen, Hungary; 2 Institute for Nuclear Research, Bem tér 18/c, 4026 Debrecen, Hungary; csik.attila@atomki.hu

**Keywords:** graphite polystyrene, thermal conductivity, specific heat capacity, heat treatments, microscopy

## Abstract

Nowadays, to improve quality of life, to have a more comfortable life, in internal spaces we try to maintain conditions that are free from external environmental influences. Thus, existing as well as newly built houses have adequate interiors maintaining their temperature, warming, or cooling due to the environment compensation. One way to create this is to reduce the heat loss in buildings. An option to achieve this is the application of thermal insulations. Nowadays, the use of super insulation materials such as aerogel and vacuum insulation panels and other nano-structured insulations, such as graphite doped expanded polystyrene, is becoming increasingly justified. These are relatively new materials, and we know only a little about them. This paper presents research results based on temperature-induced investigations of nanostructured graphite expanded polystyrene, to reveal its thermal stability after long-term and short-term thermal annealing, simulating the ageing of the material. Firstly, with a differential scanning calorimeter, we will explore the thermal stability profile of the specimens. After this, the paper will present temperature-induced changes in both the thermal properties and the structure of the samples. We will also present changes in the thermal conductivity, modifications in the surface, and compressive property variation induced by thermal annealing. The samples were thermal annealed at 70 °C for 6 weeks, at 100 and 110 °C for 1 h. Besides the thermal conductivity measurements with Netzsch 446 heat flow meter equipment, we will present specific heat capacity measurement results executed with the same equipment. Moreover, sorption isotherms of the as-received and annealed samples were registered and completed with hydrophobic experiments, too. Furthermore, from the measurements, we showed that temperature should affect a significant change in the thermal conductivity of materials. Moreover, the changes in the graphite expanded polystyrene before and after thermal annealing were investigated by Scanning Electron Microscopy, as well as optical microscopy. The structural changes were further followed by an X-ray diffractometer and the IR absorption capability was tested, too.

## 1. Introduction

These days, reducing the emission of greenhouse gases and the heat loss of buildings is a key point. In the European Union, energy consumption of buildings accounts for about 20–40% of total energy consumption [1,2]. A solution for this is to use thermal insulation on the walls. The necessary energy production is associated with high pollution, as well as high operational buildings. It also entails costs since the amount of energy required can be reduced [3,4,5]. With thermal insulation, we reduce both the release of the amount of heat generated in the building or introduced by the building to the external environment, or it also minimizes the amount of heat entering by radiation. Moreover, the task of the external boundary structures of buildings is to separate the interiors of the building from the natural environment and its undesirable effects on us. Their goal is to create and maintain appropriate internal environmental factors in the artificial built environment. During this process, the various effects of weather and nature appear on the external boundary structure of buildings as a damaging effect [6,7]. These effects age and wear out these materials over time and they become less effective. The situation is even more serious if these structures are not properly chosen and thus additional, non-general damaging factors occur. With laboratory circumstances, one can simulate the passage of time, and one can estimate the lifetime, long-term performance as well as degradation mechanism of the samples. Heat treatment can be a way to simulate the above-mentioned processes, artificially. Thermal annealing enhances both the chemical and physical kinetic processes within the samples. For insulation materials, the heat treatments should be performed at around 70 °C for weeks. During this ageing, processes become faster, but not as much as one expects. For this, higher temperatures (around 100 °C) can also be applied. These temperatures can elevate both the structural and the physical (thermal) properties of the materials, such as specific heat capacity and thermal conductivity; moreover, plastic foams can suffer from shrinkage. Thermal insulation materials should remain stable for 30–40 years. In real cases, in summer or on a hot day plastic insulation material can suffer heat load during the implementation, if it is not protected from sunlight [8,9,10,11,12,13,14].

Plastic insulation materials having dark colors can be heated up easily. When exposed to the scorching sun, its surface temperature quickly reaches a critical 80 degrees Celsius, where the material is already deforming and melting. Deformed insulation sheets become unusable. Technological development has brought with it the development of buildings and building structures [15]. For thermal insulation of buildings, several types of materials are used in both fibrous and cellular forms, while they can be organic or inorganic and natural or polymerized. Polystyrene foam is the most common family of thermal insulation plastics and is a material manufactured from pre-foamed styrene beads with a density of 10–40 kg/m^3^. Polystyrene foam products are expanded polystyrene beads, expanded polystyrene (EPS) powder, polystyrene foam blocks, polystyrene sheets and polystyrene foam covers [4,16,17,18,19,20,21].

### Topics of the Paper and Importance of Graphite Polystyrene

In this paper, we present thermal annealing experiments executed on graphite embedded/enhanced expanded thermoplastic cellular polystyrene (GEPS) rigid foam thermal insulation material. Its raw material is hard white produced by chemical raw material manufacturer’s pearls, a thermoplastic polymerized styrene and pentane, and a flame retardant additive. Heat exposure to polystyrene softens granulate particles due to the expansion of the foaming additive; inside they inflate. The pre-foamed particles (again by steaming) are closed and, according to their shape, they expand or stick together in space. After molding, the polystyrene elements shrink by approximately 90%, so this should be taken into account in applications. Graphite thermal insulation has significantly better thermal insulation capability than conventional (white) products. It is based on polystyrene and microscopically small graphite contaminants as an additive during the polymerization, thus improving the insulation [22,23,24,25,26]. The graphite additive reduces the radiant heat transfer in the polystyrene cells and thus the material’s thermal conductivity will be more favorable. Expanded polystyrene foam insulation materials allow effective thermal insulation of building structures. The material is economical and is currently available for the least amount of money. Graphite polystyrene foam sheets, which have an approximately 20% lower thermal conductivity than graphite-free EPS sheets, are successfully used in the construction industry due to the addition of microscale graphite powder [27]. In addition to the pentane gasifier, micro-graphite powder is added to the polystyrene during the production of the expanded cellular products. In expanded polystyrene insulation boards, closed air cells separated by thin walls provide thermal insulation. In the graphite-doped EPS plate, the infrared heat radiation is greatly reduced by the radiation-absorbing and reflective graphite powder particles on the cell wall. On the nanosized graphite particles acting as a heat mirror, the heat radiation is scattered and partially reflected, thereby increasing the thermal conductivity of the thermal insulation material [28,29]. This paper presents research results based on temperature-induced investigations of nanostructured graphite expanded polystyrene, to reveal its thermal stability after long-term and short-term thermal annealing. Firstly, with a DSC 3500 Sirius differential scanning calorimeter, we explored the thermal stability profile of the specimens. We also represent changes in the thermal conductivity, modifications in the surface, and compressive property variations induced by thermal annealing. The samples were aged through thermal annealing at 70 °C for 6 weeks and at 100 and 110 °C for 1 h. Besides the thermal conductivity measurements with Netzsch heat flow meter (HFM) 446 s equipment, the article presents specific heat capacity measurement results executed with the same equipment. Moreover, sorption isotherms of the as-received and annealed samples were registered and completed with hydrophobic experiments. Moreover, the changes in the graphite expanded polystyrene before and after thermal annealing were investigated by scanning electron microscopy (SEM), as well as with optical microscopy. We revealed the shrinkage process; moreover, the structural changes were further observed by an X-ray diffractometer (XRD). We also executed IR absorption tests. These comprehensive investigations are important from the point of view of ageing the materials, because graphite enhanced polystyrene is a common and relatively new material. Our results can be important for both designers and researchers.

## 2. Materials and Methods

### 2.1. The Thermal Conductivity of Insulation Materials

It is a general fact that in a cellular thermal insulation product, the heat distribution and the effective thermal conductivity (λ_eff_, W/mK) are divided into four terms: for the conduction through the solid (λ_c_) describing the heat conduction of the gas (λ_g,c_) and the members defining its gas convection (λ_conv_), while λ_rad_ belongs to the radiation. The process is presented with Equation (1), [4,9,30]:

λ_eff_ = λ_c_ + λ_conv_ + λ_g,c_ + λ_rad_. (1)

**Figure 1:**
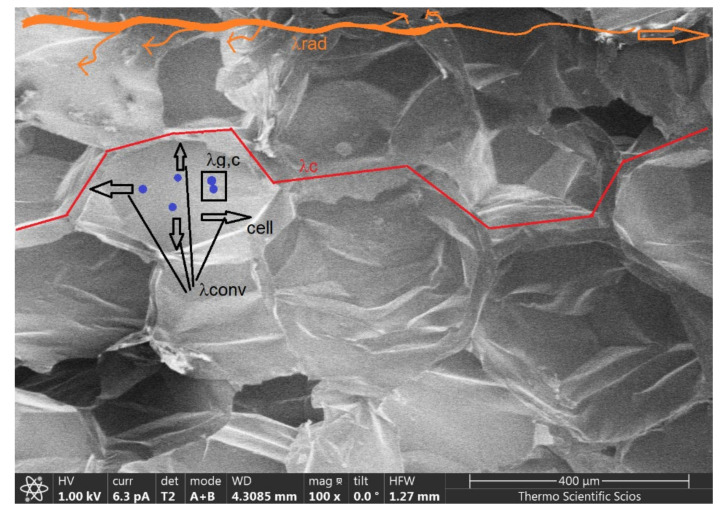
The heat transfer inside the graphite EPS.

For porous materials, the convective part goes to zero with high density (decreasing pore size) and will be negligible; moreover, increasing the absorption coefficient of materials at a given temperature (by adding a light-absorbing or reflective additive) is an effective way to suppress the thermal conductivity, such as the addition of carbon/graphite particles to polystyrene to further reduce the effective (total) thermal conductivity (see Figure 1).

Figure 1 represents the heat transfer mechanisms inside the graphite EPS material. One can see the conduction and the convection of the gas inside the cells, the conduction through the connected cell walls as well as the radiation and the weakening (reflection) of the radiation through the sample, caused by the graphite particles [27].

### 2.2. Tested Materials

In this paper, we present multiscale thermal investigations executed on graphite enhanced expanded polystyrene insulation, with an initial density of approx. 17 kg/m^3^, having a 30 × 30 cm base area and approximately 4.5 cm thicknesses.

### 2.3. Thermal Characterization with Netzsch DSC Sirius 3500

Thermal characterization of the materials was executed by a Netzsch differential scanning calorimeter (DSC) type Sirius 3500. Two types of measurement were executed. Firstly, to reveal the heat-induced changes a sample with 10 mg weight was placed in an alumina crucible, and the thermal profile was registered from 20 to 150 °C of with 10 K/min heating rate under nitrogen flow. Secondly, to find the specific heat capacity (Cp, J/kgK) of the samples changing with the temperature, a three step method was executed with the DSC, such as the baseline correction with a sapphire reference and the sample, under the same conditions of each. From the heat flow data, two standard methods were used to perform the Cp, namely the DIN 51,007 and the ratio method (ASTM E 1269) [31,32,33].

### 2.4. Structural Investigations with Microscopic Techniques (SEM, OPTICAL)

To reveal the structure of both the as-received and annealed samples, microscopy images were taken. Images from the bulk samples were taken both with a camera and an optical microscope with 20× and 50× magnification. To visualize the cell structure of the samples, scanning electron microscope images were taken. The surface morphology of the samples was investigated with a dual-beam scanning electron microscopy type Thermo Fisher Scientific-Scios 2 (FIB-SEM, Waltham, MA, USA) operated at a low accelerating voltage (1 keV). Applying such low energy and short working distance allows us to study the surface morphology of insulating samples without coating them with a gold layer. The advantages of low acceleration can result in a lower penetration depth of the accelerated electrons which results in a decrease in the volume of the interaction [34]. The secondary electrons generated near the surface in the reduced value can easily escape, which increases the secondary electron yield. As a consequence of low acceleration voltage, it can be achieved that the specimen current will be equal to zero because the incoming and outgoing currents will be the same. This means that no electric conductivity of the specimen is required to eliminate the charge accumulation and insulation materials can be studied without applying a conductive coating layer, which may modify the morphology of the surface.

In addition to scanning electron microscopy, the surface of the samples was also examined on a larger scale using optical microscopy. The Keyence VHX-6000 digital 3D microscope was applied for imaging.

### 2.5. Thermal Conductivity and Specific Heat Capacity Measurements with Netzsch 446 HFM

Both the specific heat capacity and the thermal conductivity of the as-received and thermally annealed (bulk) samples were tested with a heat flow meter namely the Netzsch 446 s equipment. The thermal conductivity tests were accomplished according to standards ISO 8301 and 10456 [35,36]. Both the thermal conductivity and specific heat capacity measurements were executed on samples having a 20 cm × 20 cm base area, the samples for the tests with the HFM were cut out. The details of the measurement method are presented in the latest papers of the authors [33,37]. By applying this equipment, less than 5% accuracy in the results can be expected.

### 2.6. Wetting Experiments

#### Sorption Isotherm Measurements

Wetting properties of the insulation materials, such as the surface wettability and the sorption isotherms, are key characteristics from the applicability point of view. Both the untreated and the treated samples were cut up and their sorption isotherms were registered according to the standard ISO 12571 [38,39]. For the registration of the sorption isotherms from 30% to 90% relative humidity and under 23 °C, a combination of drying equipment such as Venticell 111 and a climatic chamber (Climacell 111) were completed and a milligram preciseness balance was used. Moreover, the surface wettability of the samples was also tested and the contact angles of a water droplet on the surface were evaluated with ImageJ software. We can consider the material hydrophobic if the contact angle between the surface and the water droplet is greater than 90°, while it is hydrophilic if the contact angle is less than 90° [9,21,37].

### 2.7. X-ray Diffractometry Analysis

To obtain crystallographic information from the as-prepared and annealed samples the X-ray diffraction (XRD) measurements were performed by a Rigaku SmartLab diffractometer using CuK-alpha irradiation with wavelength CuKα = 0.154 nm. Scans were performed in theta–2theta scanning geometry and the X-ray tube was operated with 200 mA and 45 kV. Diffraction patterns were measured between 10° and 100° to study the possible formation of the crystalline phase in the samples. Before the measurement, a piece of graphite expanded polystyrene was cut with a sharp knife and the measurement was performed on a prepared flat surface.

### 2.8. The Tested Material

The tested material is a common and widely used graphite enhanced insulation slab. The grey color of the material is due to the addition of ultra-fine-grained graphite, which improves its thermal insulation by 20% compared to standard white sheets. We have collected the declared properties of the graphite EPS in tablet form (see Table 1).

Here it has to be mentioned that the first annealing temperature was set to 70 °C under the reported stability temperature by the manufacturer. Moreover, let us mention that our material was not covered with a light-absorbing paint layer on the surface.

## 3. Results and Discussion

### 3.1. Results of the Differentiated Scanning Calorimetry Experiments

It is well known that DSC is a perfect tool for revealing the thermal properties of solid materials, specifically polymers or plastics [25,33,40,41,42]. DSC studies can be used to monitor the various thermodynamic and relaxation transitions in plastics. Amorphous polymers have a characteristic relaxation transition: glass transition. At glass transition temperature, the segment movement is released (heating) or freezes (cooling). The appearance or disappearance of a new form of molecular motion is accompanied by a change in the specific heat capacity (Cp), which appears as a step on the DSC curve. In most cases, in addition to the stepwise change in Cp, a local maximum can also be observed on the DSC curve. Its size increases with an increased heating rate (due to overheating).

**Figure 2:**
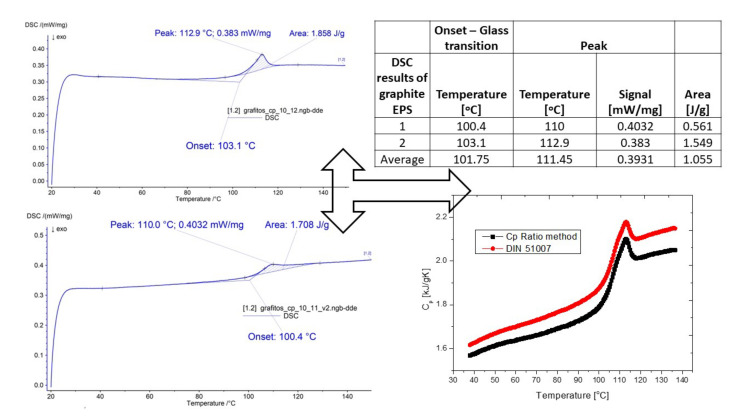
The measurement results of the graphite EPS samples with the DSC equipment.

As above presented, two types of measurements were executed. Firstly, to reveal the heat-induced changes a sample with a 10 mg weight was placed in an alumina crucible, and the thermal profile (heat flow) was registered from 20 to 150 °C with a 10 K/min heating rate under nitrogen flow. Two measurements steps were accomplished and their average was calculated. The reason for this test was to find suitable temperatures for the heat treatments (ageing). In Figure 2 (left-hand side), one can see the heat flow versus the temperature profiles. The glass transition temperature (T_g_) is assigned to the onset at about 100 °C (see Figure 2). Moreover, a peak was registered at about 110 °C. By using both the ratio and the DIN method the Cp versus temperature curves were found. From the curves, we can conclude that the oxidation of the sample is expectable. These temperature values (100 and 110 °C) designated the ageing (heat-treatment) temperatures of the bulk samples. It is presented that the glass transition temperature of the pure white EPS can also be found at about 95–100 °C, as well as that of the glass transformation of PS macromolecules chains [43,44].

### 3.2. Structural Changes in the Samples after Thermal Annealing Scanning and Optical Microscopy Measurement Results

**Figure 3:**
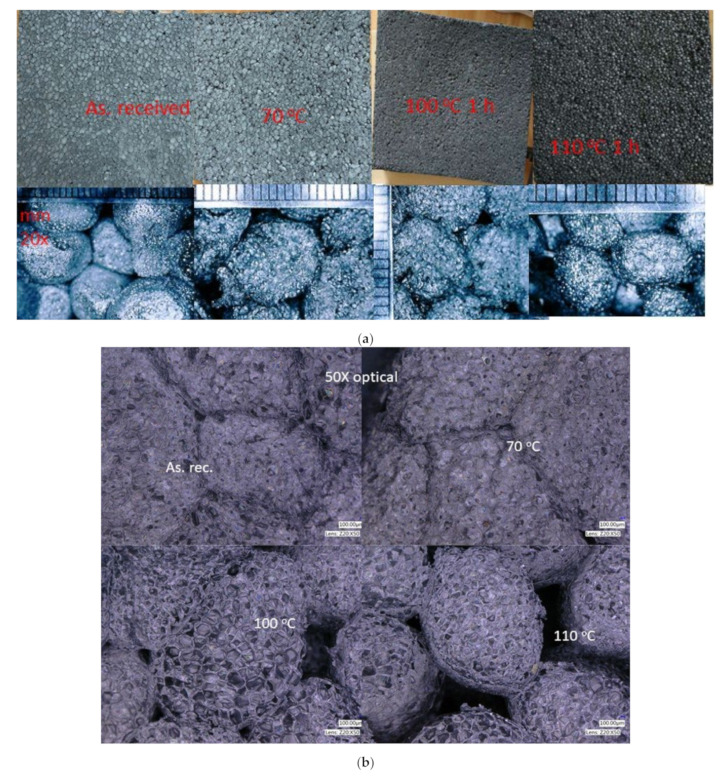

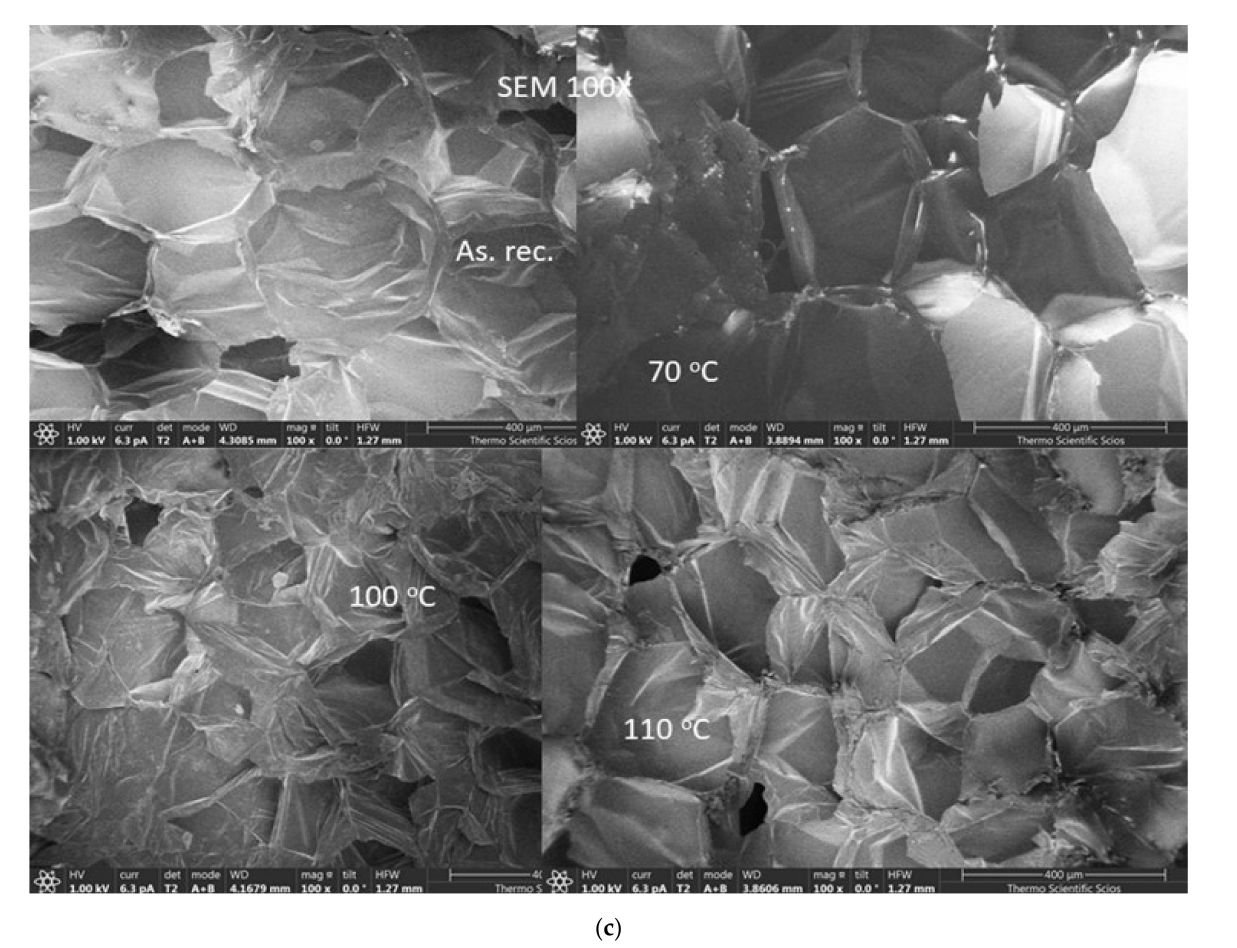
(**a**) Photo images from the samples (from left to right: As received, annealed at 70 °C, annealed at 100 °C and annealed at 110 °C). (**b**) Optical microscope images from the samples. (**c**) Scanning Electron microscope images from the samples.

The DSC results designated the temperatures for the thermal annealing. So, the samples were aged through thermal annealing at 70 °C for 6 weeks and at 100 and 110 °C for 1 h. The reason for the long-term thermal treatment was the following. Initially, we have heat-treated samples at 70 °C for 1 day, and we did not see any changes in them. As is well known, the thermal treatment of polymeric samples should be executed near 70 °C for weeks. So, we have thermally treated the samples for 6 weeks at 70 °C, moreover, the dimensional stability temperature of the sample is also 70 °C. Furthermore, from DSC profiles we have seen that, at 100 and 110 °C, a 1h heat treatment should be enough to reveal the degradation process. In Figure 3a–c, one can see photo images and optical microscope images as well as SEM images from all the samples. From Figure 3a one can see that after the long-term heat treatments significant change did not happen, while after the short-term thermal treatments (at 100 and 110 °C) the samples deformed, and at 110 °C the surface visibly changed. This is further stated in Figure 3b,c because one can observe shrinkage and distancing of the beads. Furthermore, from Figure 3b one can see that the macroporosity (gap among the beads) is increasing after thermal annealing the samples at 100 and 110 °C; it is also visible in Figure 3c [45]. Moreover, from Figure 3c an average cell diameter can be estimated for the samples, such as about 280 μm for the as-received and the annealed sample at 70 °C, while for the samples annealed at 100 and 110 °C 185 μm and 170 μm can be measured respectively with about ±10% accuracy.

From the measurements of the densities of the bulk samples before and after their heat treatments, we deduced a change in it, as shown in Table 2. The increase in the densities mainly comes from the shrinkage of the whole sample. We have to mention that the samples suffered a significant change in their base area and thickness after the heat treatments at 100 and 110 °C caused by the melting. Finally, the density increased by two and a half times the initial value. As was declared by the manufacturer, we revealed that the geometry of the insulation slab remains constant at 70 °C, while the geometry changed significantly from 30 cm × 30 cm × 4.5 cm to 24.8 cm × 26 cm × 4,15 cm as well as to 21 cm × 21 cm × 3 cm, belonging to the annealing temperatures 100 and 110 °C, respectively. As is known, the PS foam would shrink above 90 degrees C due to the glass transformation of PS macromolecules chains. We recorded the shrinkage size and the beads’ size distribution of GEPS sheets.

**Figure 4:**
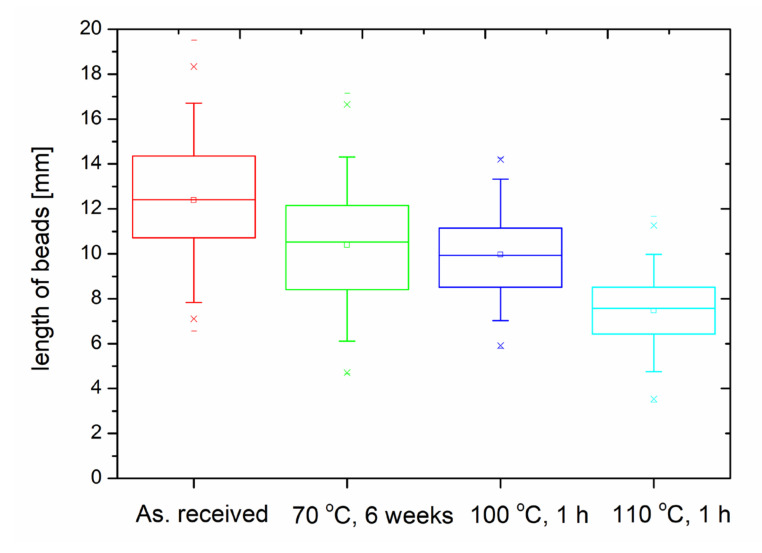
The results of the beads analysis with the box chart method.

After these microscopy experiments, beads were separated from the bulk samples and their length was measured (in mm) with an optical microscope under 20× magnification. On these raw data, statistical analysis was executed, with a box chart method. Figure 4 presents the results of the box chart analysis. One can observe that the average length of the beads is decreasing with the increasing temperature of the thermal treatment. It falls from about 12.5 mm to about 8 mm after annealing the samples at 110 °C.

**Figure 5:**
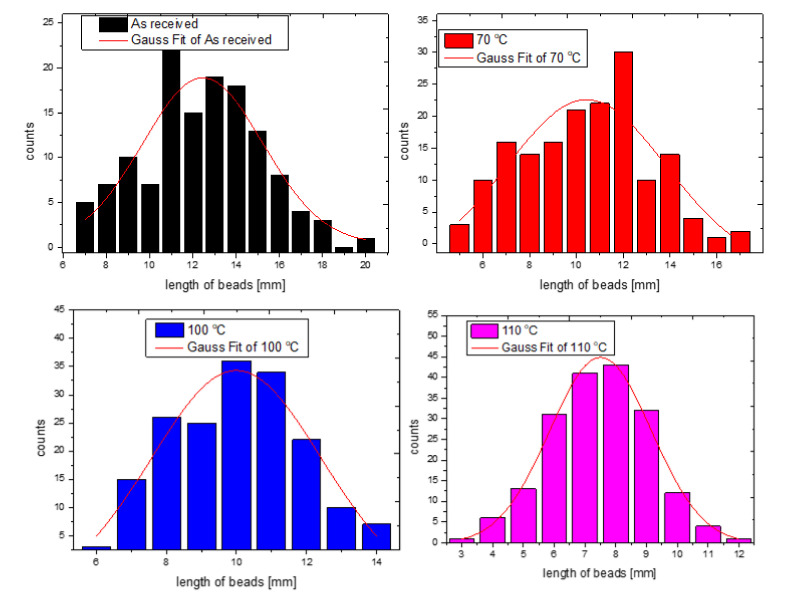
The results of the beads analysis with box chart method.

Moreover, on the size of the GEPS beads, a Gaussian distribution model was also applied and it is presented in Figure 5.

The results of the Gaussian distribution analysis are also presented in Table 3. The model used a built-in module of the data analyzing software. The equation of the model is given as Equation (2).

y = y_o_ + (A/(w·sqrt(Pi/2))) × exp(−2((x − x_c_)/w)^^2^, (2)

where w is the width, A is the area, y_o_ = 0 is the offset, x_c_ = is the center, and sigma is half of the width as the standard deviation.

From Table 3 one can see that the Gaussian distribution also states the continuous decrease in the length of the beads after thermal annealing.

### 3.3. Results of the Experiments with the Netzsch 446 HFM Equipment

#### 3.3.1. Thermal Conductivity

**Figure 6:**
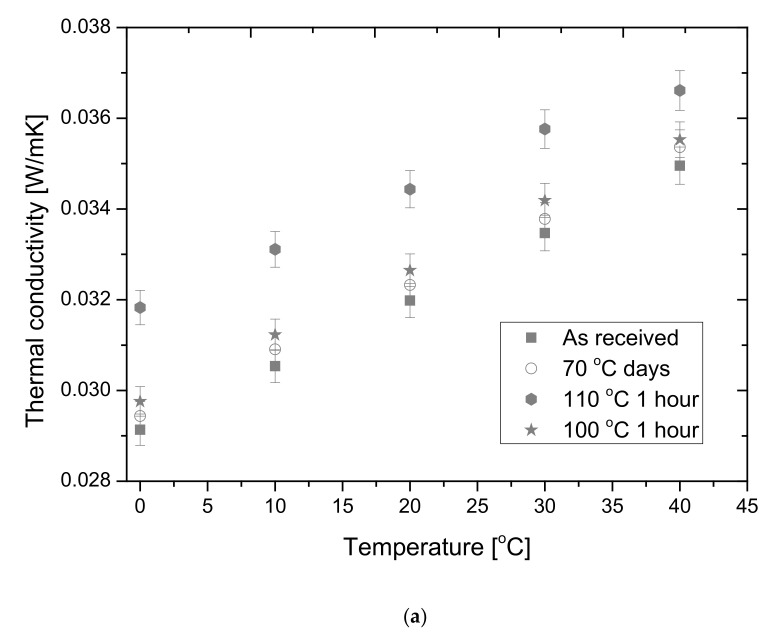

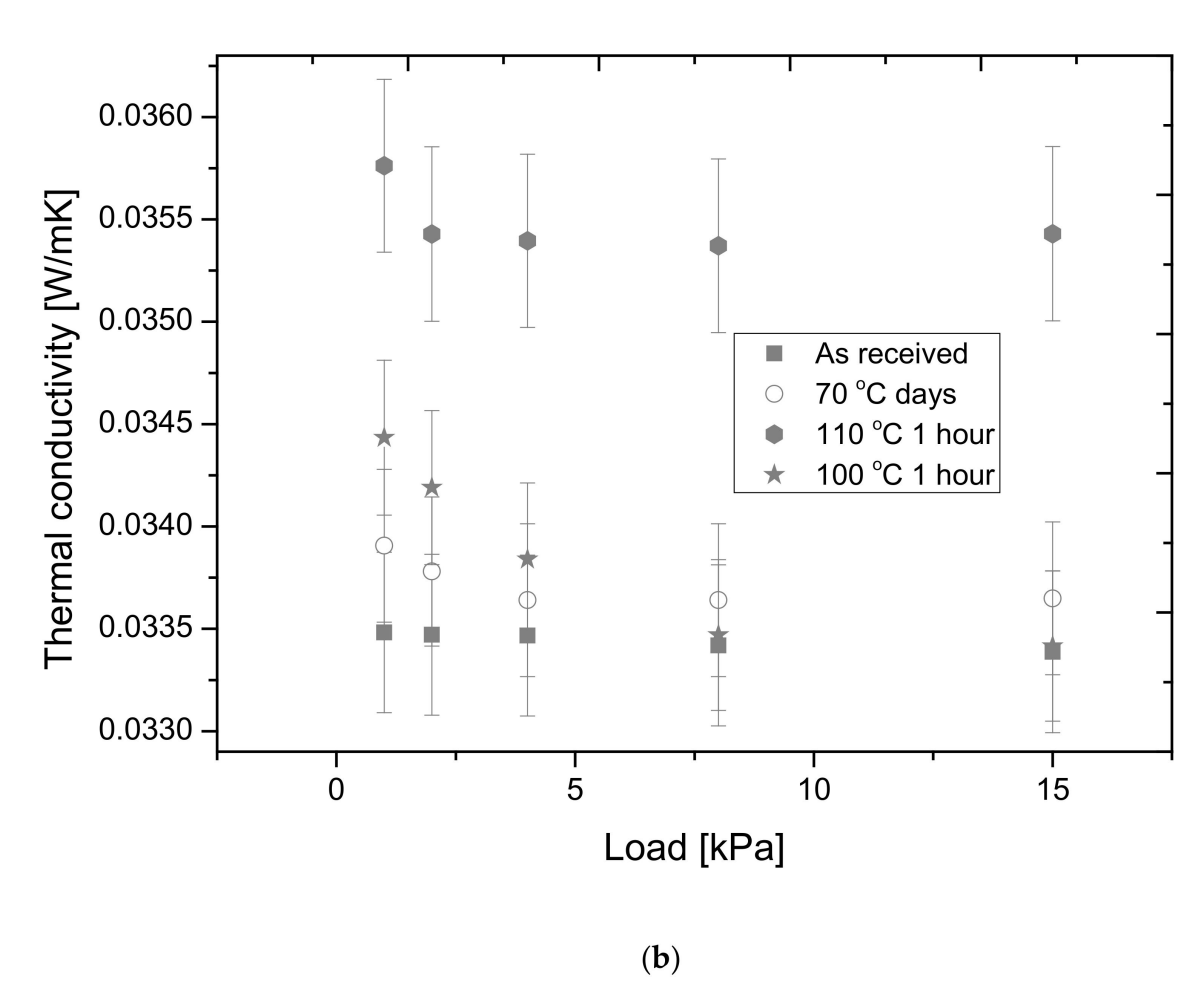
(**a**) Thermal conductivities of the samples in the function of the mean temperature. (**b**) The thermal conductivities in the function of the applied load.

The thermal conductivities of both the as-received (not annealed) and heat-treated samples were measured with the Netzsch 446 heat flow meter. Firstly, the mean temperature was set between 0 and 40 °C with 10 °C step, suffering a 20 °C temperature difference between the plates of each. From Figure 6a, one can see that the thermal conductivity is stable in the cases of thermal treatments at 70 °C for 6 weeks and 100 °C for 1 h.

We can conclude that the above presented thermo-physical changes do not cause significant changes in the thermal conductivity. After thermal annealing at 110 °C for 1 h the thermal conductivities change by about 8–10%. The thermal conductivity of material strongly depends on its density as is stated in Ref. [46]. With the shrinkage of the samples, the macroporosity of it is increasing in parallel, whit the increase in the conduction through the solid part opening to both the convection and convection of gas in the macro-pores, moreover the oxidation process further enhances the increase in the thermal conductivity. Here it has to be mentioned that these measurements stated that not only the as received but also the annealed graphite EPS insulation has a much smaller thermal conductivity than the conventional white EPS 80, which is about 0.039 W/mK measured at a 30 °C mean temperature [46].

Netzsch HFM allows the measurement of the thermal conductivities of the samples under compression loads, between 0.5 and 15 kPa. This measurement row represents the changes in the thermal conductivity under compression. Since we have measured the thermal conductivity at 30 °C mean temperature with 20 °C difference, under 1, 4, 8 and 15 kPa loads. One can see that the thermal conductivity versus load curves follows a hyperbolic shape, with a minimum, except for the as-received one (see Figure 6b). A similar effect for the change in the thermal conductivity in the function of the load was presented earlier [37].

#### 3.3.2. Changes in the Compressive Behavior of Samples

**Figure 7:**
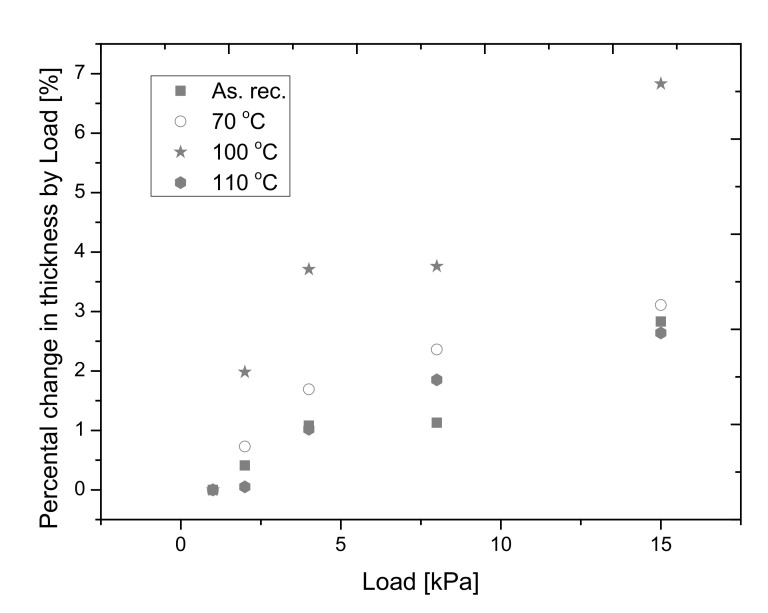
The changes in the thickness in the function of the applied load.

The declared compressive strength of the sample by the manufacturer is 80 kPa, which is an average value among the 30–200 kPa compressive strength for common EPS insulations. The Netzsch 446 hfm equipment measures the change in the thickness with an in-built caliper by the applied load [37]. In Figure 7 we represent the percental changes in the thickness. We can state that after thermal annealing at 70 and 100 °C the compressibility of the samples is increasing because the percental change in the thickness is increasing with the increasing load, so this means that the samples become softer, especially after thermal annealing at 100 °C. By applying a 15 kPa load the thickness of the sample annealed at 100 °C changes by about 7%. It is worth mentioning that after the thermal treatment of the samples at 110 °C they become rigid again and their compressibility is near the value of the un-annealed sample.

#### 3.3.3. Specific Heat Test

**Figure 8:**
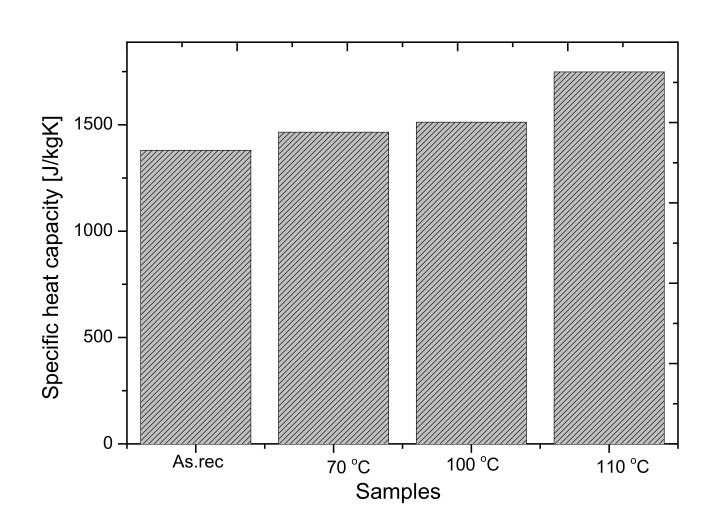
The specific heat capacities of the samples at 25 °C.

Additionally, by using the NETZSCH 446 hfm equipment, the specific heat capacities of the bulk samples were also measured. The results show that the Cp (J/kgK) value of the samples after annealing increases what can be caused by the above stated thermo-physical changes (glass transition, possible oxidation, density change). The specific heat capacity of the bulk sample slightly (<10%) changes at 70 °C and 100 °C, while at 110 °C the changes are nearly 30%. During the Cp measurements, the plates are held at the same temperature. If the constant temperature is reached, the heat flux becomes zero and the Cp value can be estimated as a step-by-step method. The temperature set was fixed to 15 °C until 35 °C and the result is given at 25 °C and is represented in Figure 8.

### 3.4. Results of the Wetting Experiments, Sorption Isotherms and Contact Angle

**Figure 9:**
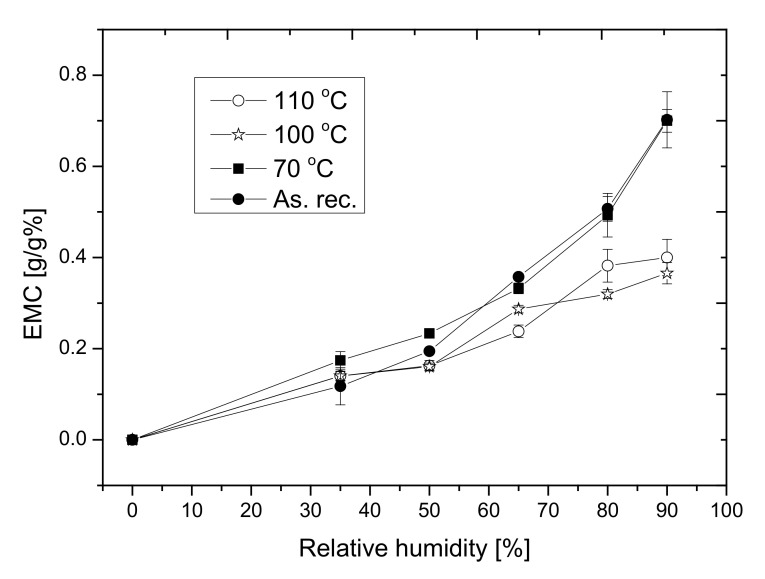
The sorption isotherms.

To reveal the changes in the surface and the pore-structure of the materials after annealing, the sorption isotherms, as well as the contact angles of a water droplet of the samples, were registered. Sorption isotherms provide information about the pore’s structure and the capillarity, while contact angle measurement results provide information on the surface change. Firstly, the above-mentioned samples having a 20 cm × 20 cm base area were cut into four pieces having a 10 cm × 10 cm surface. The sorption isotherms of four pieces of each sample were registered as the rules of ISO 12571 [38] at 23 °C and under 30–90% relative humidity. From the sorption isotherms (equilibrium moisture content (EMC) versus relative humidity graphs) represented in Figure 9, one can see that the curves of the as-received and annealed samples at 70 °C go together and they follow Brunauer type III isotherms, which forecasts a strong cohesion force between adsorbed molecules if the sample is hydrophobic. Moreover, the adsorption affinity decreases after heat treatments at 100 and 110 °C. It is mainly caused by the change of the surface structure, especially in the damage of the cell structure and the distancing of beads [47]. In the graph besides the measured value, the estimated errors are also presented.

**Figure 10:**
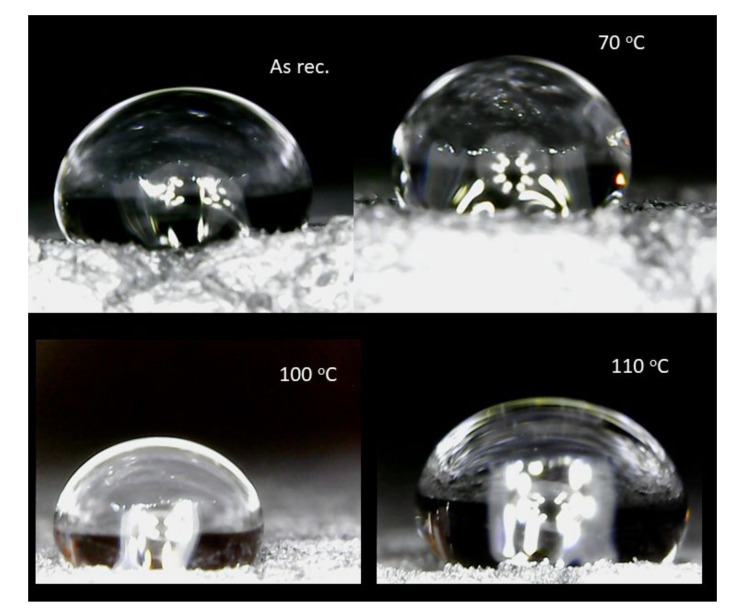
The hydrophobic test.

Contact angle measurements were executed to further understand the possible surface modifications. Table 4 as well as Figure 10 present the results of the contact angle measurements. A water droplet was positioned with a pipette on the surface of the samples, and a photo image was taken from them. The images were evaluated with the ImageJ software.

The results show that the contact angle is slightly increasing after thermal annealing. With this, we can further support a modification in the surface of the sample after thermal annealing, which could also entail internal structural changes.

### 3.5. Results of X-ray Diffraction Measurements

**Figure 11:**
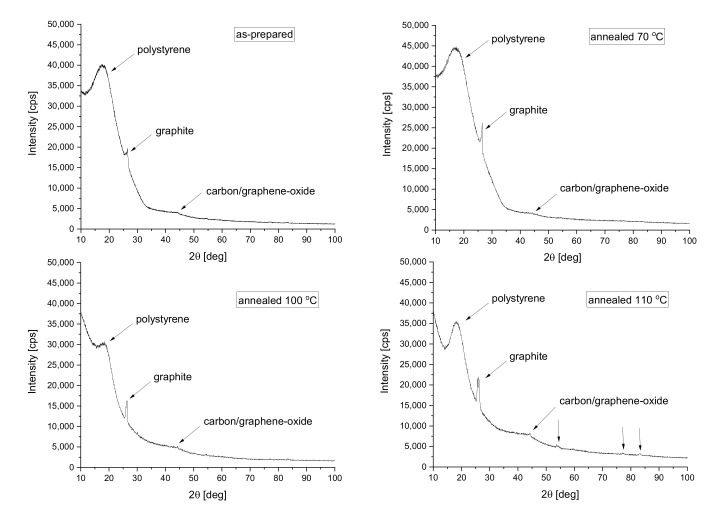
The X-ray diffractometer results.

Finally, to reveal and justify the above-stated process, the paper presents X-ray diffractometer analysis results executed on the samples. Figure 11 presents the results of X-ray diffraction measurements performed in theta-2theta geometry.

Near 20°, a wide broad peak was observed, which was attributed to the amorphous polystyrene beads. One can see that the peak keeps its position and remains broad, confirming the presence of the amorphous structure in all measured samples [48,49,50]. We can also further state that at 26.4° another significant diffraction was observed belonging to the graphite (according to ICDD database PDF card number #00-056-0159) [51,52,53], while at about 43.7° the strongest line of graphene-oxide (carbon-oxide; according to ICDD database PDF card number #01-082-8807) is visible. It can be seen that the intensity of this peak increased slightly with increasing annealing temperature and additional peaks, related to the oxide phase have also appeared in the range above 50 degrees. This indicates that the proportion of oxidized graphite in the material increases with increasing heat treatment temperature. Furthermore, from the point of view of thermal conductivity, we can say that the oxidation process of the graphite can also cause a reduction in its good thermal reflective property. The compressive properties of the samples changed as well.

### 3.6. Infrared Absorption Test

**Figure 12:**
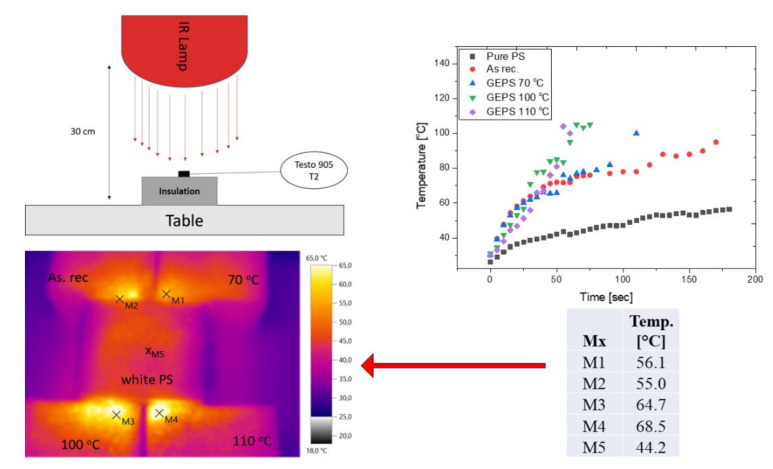
IR absorption test.

Following the directions of Pan et al. in we tested the infrared absorption of the samples, with a Philips short-wave IR lamp that had 100 W as a source [54,55]. The four graphite samples and pure white polystyrene samples from the same manufacturer had the same compressive strength (80 kPa) and were positioned under the lamp at a fixed distance. The measurement order was based on the one presented by Pan and his colleagues in Ref. [55]. A Testo 883 type thermo-camera was also used to better accomplish the experiment. The measurement order and the results of the test are visible in Figure 12. Two measurement rows were executed once, when the samples were radiated with the lamp alone, one after the other and during the heating, the temperature of the surface was registered. Secondly, all the samples were placed under the lamp together, and the white one was placed in the middle (see thermograph image in Figure 12).

The individual measurements showed that the annealed samples warmed up faster and reached a greater temperature (>100 °C) compared to the un-annealed ones. Moreover, we have tested a pure (white) EPS also where the warming up was much slower and the temperatures were lower, too. These results show a good correlation with the results presented by Pan [53].

## 4. Conclusions

Laboratory tests executed on thermal insulation materials are a key point from both designing and scientific points of view. This paper presents measurement results on the artificial ageing of graphite enhanced polystyrene thermal insulation. From the results, we have stated that the graphite enhanced polystyrene is a stable insulation until 70 °C, while over this temperature both the structure and thermal properties of the material suffer from change. With a combination of different types of equipment (heat flow meter, differential scanning calorimetry and microscopic techniques), we revealed that the changes were particularly noticeable. We have revealed that after thermal treatments of the samples their density changed, caused by the shrinkage of the samples. Another interesting and visible change was that after annealing the macroporosity of the samples at 100 and 110 °C is increased, and the distancing of the beads was also observable. Moreover, the size of the beads decreased. We have pointed out the change in the thermal conductivity (with 8–10%), caused by the structural changes and the oxidation processes, especially for the sample annealed at 110 °C. Interestingly, it was also visible from the specific heat capacity measurements. Besides the thermal and structural properties, we have also deduced changes in the wetting properties of the sample. After heat-treatment of the samples, the surface hydrophobicity slightly changed parallel with the decrease of the moisture adsorption affinity. Moreover, we also tested the IR absorption property of the samples, which showed interesting results.

## Figures and Tables

**Table 1:** The declared values of the tested graphite EPS by the manufacturer.

Property	Value
Thermal conductivity at 10 °C mean	0.032 W/mK
Dimensional stability	at 70 °C it is 3%
Compressive stress at 10% compression	80 kPa
Components	C, O, H

**Table 2:** The measured densities of the samples.

Sample	Density [kg/m^3^]
As received	16.83
70 °C	16.51
100 °C	23.27
110 °C	43.21

**Table 3:** The results of the Gaussian fits.

	x_c_	x_c_	w	w	Sigma	FWHM
	Value	Standard Error	Value	Standard Error	Value	Value
As received	12.45	0.29	5.55	1.17	2.77	6.54
70 °C	10.43	0.38	6.79	2.95	3.39	8.00
100 °C	9.99	0.1736	4.83	1.71	2.41	5.68
110 °C	7.5	0.054	3.30	0.18	1.65	3.89

**Table 4:** The measured contact angles.

Sample	Contact Angle [°]	Est. Err. (±)
As received	109.67	2.22
70 °C	110.83	1.55
100 °C	111.67	3.05
110 °C	112.17	3.38

## Data Availability

Not applicable.
